# Controlling the symmetry of inorganic ionic nanofilms with optical chirality

**DOI:** 10.1038/s41467-020-18869-9

**Published:** 2020-10-14

**Authors:** Christopher Kelly, Donald A. MacLaren, Katie McKay, Anthony McFarlane, Affar S. Karimullah, Nikolaj Gadegaard, Laurence D. Barron, Sonja Franke-Arnold, Frances Crimin, Jörg B. Götte, Stephen M. Barnett, Malcolm Kadodwala

**Affiliations:** 1 School of Chemistry, University of Glasgow, Glasgow, G12 8QQ UK; 2 SUPA, School of Physics and Astronomy, University of Glasgow, Glasgow, G12 8QQ UK; 3 School of Engineering, Rankine Building, University of Glasgow, Glasgow, G12 8LT UK; 4 College of Engineering and Applied Sciences, Nanjing University, Nanjing, 210093 China

**Keywords:** Chemical physics, Nanophotonics and plasmonics

## Abstract

Manipulating symmetry environments of metal ions to control functional properties is a fundamental concept of chemistry. For example, lattice strain enables control of symmetry in solids through a change in the nuclear positions surrounding a metal centre. Light–matter interactions can also induce strain but providing dynamic symmetry control is restricted to specific materials under intense laser illumination. Here, we show how effective chemical symmetry can be tuned by creating a symmetry-breaking rotational bulk polarisation in the electronic charge distribution surrounding a metal centre, which we term a meta-crystal field. The effect arises from an interface-mediated transfer of optical spin from a chiral light beam to produce an electronic torque that replicates the effect of strain created by high pressures. Since the phenomenon does not rely on a physical rearrangement of nuclear positions, material constraints are lifted, thus providing a generic and fully reversible method of manipulating effective symmetry in solids.

## Abstract (web summary)

The symmetry of metal ions in inorganic nanofilms can be manipulated by the transfer of optical spin from a chiral light beam. Here the authors present a route to functional manipulation that does not require the application of extreme conditions.

## Introduction

The symmetry environment of metal centres in compounds defines many of their functional properties. Dynamic control of material symmetry consequently allows time varying switching of properties required for device applications^1^. Lattice strain is the most natural agent for tuning symmetry properties since it physically alters the relative positions of constituent chemical species. Light–matter interactions have been used to provide a controllable means of introducing strain into certain materials. They can induce strains that would otherwise require extreme external forces, and they can enable states of matter to be reached that are inaccessible with adiabatic thermodynamic processes^2,3^. However, a fundamental weakness of using such light–matter interactions is that they are non-generic, relying on a material-specific property, such as the presence of either a phase transition^2–6^ or weakly bound layers^7^ in crystalline solids. This greatly limits the range of materials that can be optically tuned and hence restricts potential applications.

Circularly polarised light (CPL) can induce symmetry reduction in chemical environments of photoactive materials^8–11^, an ability that is associated with its inherent sense of handedness, a property known as chirality, conveyed by its intrinsic spin angular momentum. Effectively the inherent sense of twist of the spin angular momentum of the CPL is transferred to the physical structure, breaking mirror symmetry. However, conventionally CPL can only act as a symmetry-reducing influence in photoactive systems, which either have a chiral component or have the potential to be chiral. There has been no previous evidence that illuminating an intrinsically non-chiral photo-inactive material with CPL can alter the symmetry properties of the chemical environments of the constituent species. In contrast to atomic dimensions, transfer of optical spin angular momentum can impart a sense of physical twist on a much longer length scale. Angular momentum transfer from CPL has been used in opto-mechanics to impart torques to rotate mesoscale objects about the incident beam axis^12–15^. However, the torques are far too weak to break chemical symmetry by physically distorting chemical bonds. Consequently, the potential of chiral light for symmetry breaking in materials that have no chiral character has never been established.

Here we demonstrate a chiral light–matter effect for reversibly manipulating the effective symmetry of chemical environments within a solid material, without requiring a physical distortion of the chemical structure. We propose that the effect arises from a transfer of optical spin angular momentum from a chiral light beam through an interfacial mechanism. The mechanism involves the transfer of optical spin angular momentum to create an “electronic torque” that distorts the electron density surrounding a metal centre. The resulting bulk polarisation, which circulates around the incident beam, acts as a symmetry-reducing perturbation on the local chemical environment. By analogy with the crystal field established by ligands around a coordinated metal ion, the bulk polarisation effectively acts as a “meta-crystal field” to create an electrostatic potential that acts as a second-order perturbation on the symmetry environment of the metal centres. This effect is considered to be generic for ionic inorganic films containing transition and lanthanide metal ion centres.

Here, we establish the efficacy of the phenomenon using 150-nm-thick Eu_2_O_3_ nanofilms deposited on a transparent polycarbonate substrate. Eu_2_O_3_ films were chosen because Eu^3+^ is exquisitely sensitive to its co-ordination environment and the symmetry-reducing effects of the meta-crystal field can be detected in-situ using luminescence spectroscopy. The sole role of the Eu^3+^ is that of a spectator probe ion and it is not specifically necessary for the creation of the meta-crystal field. In a second experiment, we use chiral nanostructures to modulate the amount of spin angular momentum transferred to the Eu_2_O_3_ films. In both cases, symmetry reduction is reversible because the electronic torque is removed when the light is switched off. Experimental results are complemented with numerical finite-element simulations allowing the spin transfer to be quantified directly, demonstrating this effect as the cause of changes in Eu^3+^ luminescence owing to symmetry reduction.

## Results

### Continuity equation for optical chirality

Angular momentum endows light with a handedness which for our purposes can conveniently be quantified by the optical chirality density, *C*^16–19^:

1 $$C = \frac{1}{2}\left( {\mathbf{D}} \cdot \dot{\mathbf{B}} - {\mathbf{B}} \cdot\dot{\mathbf{D}} \right),$$

where **D** is the displacement field, **B** the magnetic induction and ${\dot{\mathbf{D}}}$ and ${\dot{\mathbf{B}}}$ are their respective time-derivatives. In free space, the optical chirality density is conserved, but interfaces act as sinks and sources of chirality. In order to derive a continuity equation for the optical chirality at an interface, we introduce a complex permittivity, $\varepsilon = \varepsilon^\prime + i\varepsilon^{\prime\prime}$, and permeability, $\mu = \mu^\prime + i\mu^{\prime\prime}$, and assume that the incident light is sufficiently narrow band that *ε* and *μ* can be approximated as being independent of frequency. The real fields in Eq. (1) are also written in terms of: ${\mathbf{D}} = ( {\varepsilon {\pmb{\cal{E}}} + \varepsilon^\ast {\pmb{\cal{E}}}^\ast } )$ and ${\mathbf{B}} = \left( {\mu {\pmb{\cal{H}}} + \mu^\ast {\pmb{\cal{H}}}^\ast } \right)$. Differentiating Eq. (1) with respect to time yields the following narrow band continuity equation^20^ (see Supplementary Note 1):

2 $$\dot C - i\omega \nabla \cdot 	 \underbrace{\left[ \varepsilon^\prime \left( \pmb{\cal{E}} \times \pmb{\cal{E}}^\ast \right) + \mu^\prime \left( \pmb{\cal{H}} \times \pmb{\cal{H}}^\ast \right) \right]}_{{\text{chirality}}\ {\text{flux}}} \\ 	= i\omega ^2 \underbrace{\left( \pmb{\cal{E}} \cdot \pmb{\cal{H}}^\ast - \pmb{{\cal{H}} \cdot \pmb{\cal{E}}^\ast } \right) \left( \mu^\prime \varepsilon^{\prime\prime} + \varepsilon^\prime \mu^{\prime\prime} \right)}_{{\text{loss}}\ {\text{term}}} \\ 	\quad + i\omega \underbrace{\left[ \left( \nabla \varepsilon^\prime \right) \cdot \left( \pmb{\cal{E}} \times \pmb{\cal{E}}^\ast \right) + \left( \nabla \mu^\prime \right) \cdot \left( \pmb{\cal{H}} \times \pmb{\cal{H}}^\ast \right) \right]}_{{\text{interface-exchange}}\ {\text{term}}\ {\text{(IE)}}}.$$

The left-hand side has the typical form for a conserved quantity with the time derivative of the chirality density and the divergence of its flux. The right-hand side includes a loss term corresponding to absorption and an interface–exchange (IE) term showing how chirality is lost or generated whenever the real part of the permittivity or permeability changes. This is in contrast to the optical helicity^21^, which is conserved when the interface preserves the duality symmetry of the free electromagnetic (EM) field^22,23^. For nonmagnetic or weakly magnetic systems ∇*μ*′ can be neglected. The implication of the IE term is that for CPL propagating through a transparent planar film with two inequivalent interfaces there can be a net non-dissipative transfer of chirality to the medium. Since this transfer depends on the real part of the dielectric response, it physically manifests as a polarisation within the material, which has a sense of direction about the incident beam. Specifically, the chirality flux is proportional to the spin angular momentum of the light, and its spatial change is balanced by an effective torque embodied by the interface term, which is therefore responsible for the polarisation distortion.

### The meta-crystal field model

In the case of the Eu_2_O_3_ films described here, we propose that the polarisation derives from a collective distortion of an ensemble of O^2−^ ions, which are more polarisable than the smaller Eu^3+^ ion; this reduces the effective symmetry of the co-ordination environment of the Eu^3+^. Crystal field theory describes the lifting of the degeneracy of the electronic states of a cationic metal centre by the electrostatic field produced by its nearest-neighbour anionic co-ordination environment^24^. By analogy, the “meta-environment” of an ensemble of polarised anions creates a chiral electrostatic field that perturbs the symmetry of the metal ion. In effect, the symmetry-reducing meta-crystal field is created by an electronic torque caused by the transfer of spin angular momentum arising from the exchange of optical chirality.

**Fig. 1:**
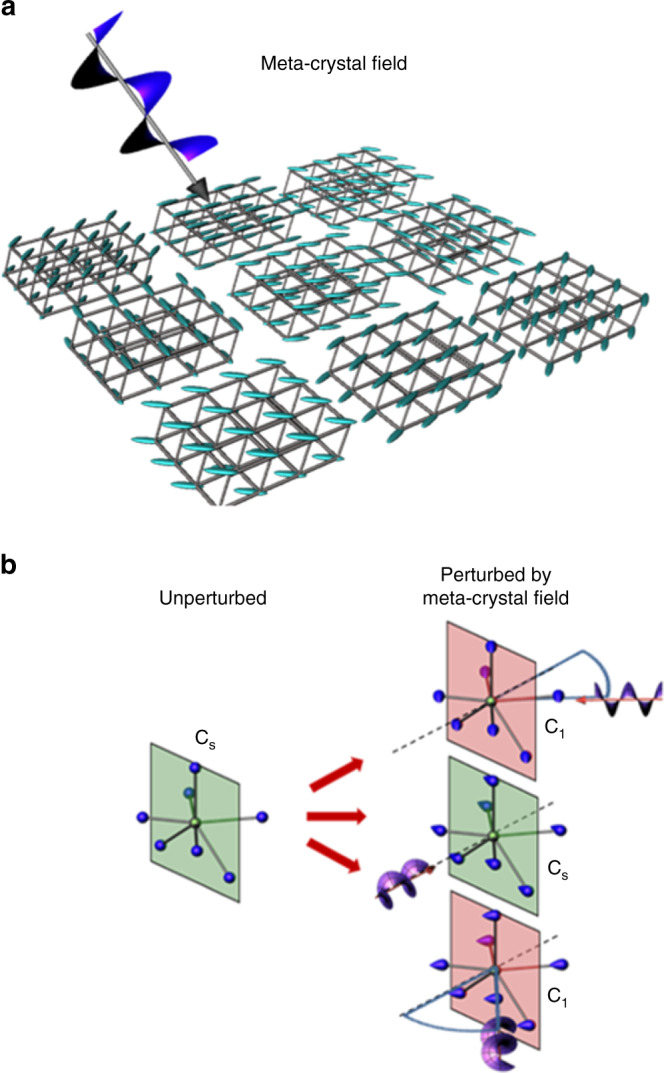
Meta-crystal fields and symmetry reduction. **a** The graphic illustrates the precession of the induced polarisation (represented by the ellipsoids) over an extended length scale. The incident CPL is shown. For simplicity the effect is illustrated with an idealised periodic lattice, the real films are structurally disordered. **b** The symmetry-reducing effects of the meta-crystal field illustrated for a monoclinic-like co-ordination environment (green and blue spheres/ellipsoids are Eu^3+^ and O^2−^ ions, respectively). A range of propagation directions relative to the mirror plane normal (dotted line) are shown. The red and green areas represent mirror planes, which have been lifted or retained, respectively, after the application of the meta-crystal field perturbation. The point group of the perturbed lattice is given beside the structure.

**Fig. 2:**
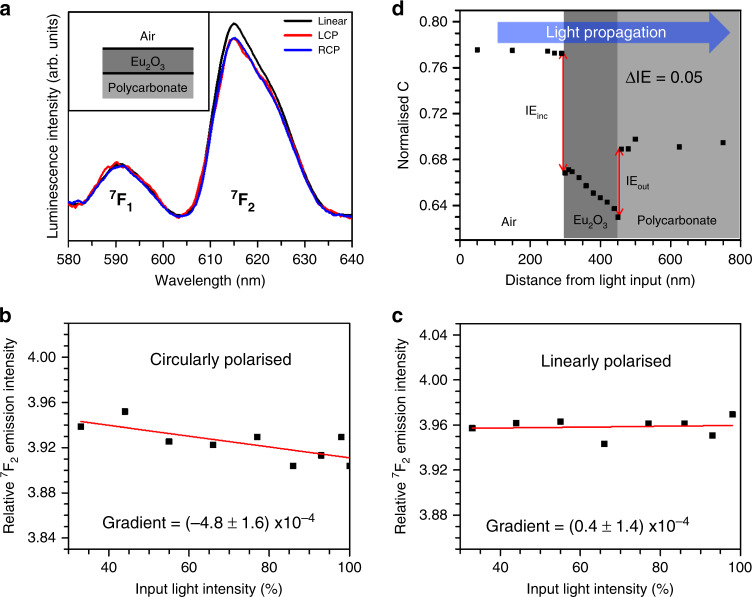
Optical chirality transfer modifies the luminescence of unstructured Eu_2_O_3_ films. **a** The relative intensity of the hypersensitive ^7^F_2_ luminescence peak decreases when illuminated with CP compared to linear light if the two interfaces (air-Eu_2_O_3_ and Eu_2_O_3_-polycarbonate in this case) have differential gradients. **b**, **c** The relative luminescence intensity decreases for CP light with input light power, but remains constant for LP light, owing to increasing optical chirality transfer in the former. **d** Numerical simulation of the change in *C* with distance from the input on passing through air, Eu_2_O_3_ and polycarbonate, using thicknesses of 300 nm (air), 150 nm (Eu_2_O_3_) and 350 nm (polycarbonate) for the three films. The magnitude of the IE terms for each interface is highlighted by the red arrow, and the net exchange of *C*, ΔIE is given.

Our proposed electronic torque is a macroscopic phenomenon, which acts over the whole irradiated area, creating an electrostatic polarisation with a local direction that precesses through 360°, Fig. 1a. On the length scale of the Eu^3+^ co-ordination environment, all the oxygen atoms would be polarised in the same direction. The induced polarisation breaks mirror symmetry, unless the incident light is orthogonal to a mirror plane of the co-ordination environment, Fig. 1b. Turning now to the data, Fig. 2a presents luminescence spectra collected from Eu_2_O_3_ films under a variety of conditions. Eu^3+^ luminescence spectra originate from *f*–*f* transitions (^5^D_0_ → ^7^F_*J*_, where *J* = 1–4). The ^5^D_0_ → ^7^F_2_ emission of Eu^3+^ ∼615 nm (subsequently denoted ^7^F_2_) is referred to as hypersensitive, being highly sensitive to the local environment of the ion compared to the other transitions^25^ (see Supplementary Fig. 1). Luminescence spectra can consequently be used to characterise the local structure of the Eu_2_O_3_ film. All of the films used show qualitatively similar luminescence spectra where the ^7^F_2_ is the most prominent band and they are consistent with the Eu^3+^ co-ordination environment being a mixture of those found in cubic and monoclinic Eu_2_O_3_^26^. The films lack long-range order and transmission electron microscopy indicates them to be amorphous (Supplementary Fig. 2). Amorphous films have significant benefits for the current study: (a) they prevent the occurrence of birefringent effects that would introduce artefacts into fluorescence measurements; and (b) they illustrate that the effect is independent of crystal structure, demonstrating its generic nature. This lack of crystallinity implies that Eu_2_O_3_ formula units will be randomly oriented throughout the film, but the local co-ordination and nearest-neighbour point-group symmetry about Eu^3+^ ions is expected to retain the co-ordination site types found in crystalline phases: C_s_ symmetry in monoclinic-like and C_2_ and S_6_, respectively, in the cubic-like environments^27,28^. Previous work has shown that Eu^3+^ ions with C_s_ and C_2_ symmetries in the monoclinic and cubic-like environments exhibit a ^7^F_2_ emission band with relatively high intensities compared to other symmetry environments^29^. Hence any reduction in local symmetry (e.g., C_s_ → C_1_) as a result of the meta-crystal field results in a decrease in the relative intensity of the ^7^F_2_ emission band.

### Luminescence and simulations of Eu_2_O_3_ films

A comparison of ^7^F_2_ emission spectra for unstructured films in air, excited by linearly polarised (LP) and left and right CPL (LCP and RCP) is shown in Fig. 2a. Since LP is not chiral, this spectrum is a reference for a non-perturbed Eu^3+^ environment. Both LCP and RCP spectra are identical and display a reduction in the relative intensity of the ^7^F_2_ emission indicative of a lowering in the effective symmetry environment of Eu^3+^. The relative intensity of CPL ^7^F_2_ emission decreases with increasing laser power, shown in Fig. 2b, and the data fit well to a straight line with a negative gradient. A linear dependence is consistent with an increasing electronic torque, produced by a greater rate of exchange of optical spin angular momentum with increasing light intensity, creating a larger symmetry-reducing perturbation. As expected for non-chiral LP light there is no dependency of relative emission intensity with power, shown in Fig. 2c. The exchange of optical chirality would be expected to be non-linear only for high intensity laser pulses when higher order terms of the electronic permittivity become significant.

Finite-element EM simulations have been performed to illustrate how *C* changes as CPL propagates through the films studied, which agree with Eq. (2). In these simulations, using COMSOL Multiphysics models of the substrate, Eu_2_O_3_, and analyte layers were created with additional integration surfaces (slices) added at regular intervals to allow *C* to be calculated with distance through the sample. The level of the symmetry-reducing perturbation is dependent only on the magnitude of the net *C* transferred and not its sign. Consequently, *C* is normalised to that of the incident polarisation, so it is always positive. From these simulations the magnitudes of the IE term in Eq. (2) can be calculated. The magnitude of the net transfer of *C* to a film (ΔIE) for an incident polarisation is defined as follows:

3 $${\rm{\Delta}}{\rm{IE}} = {\rm{IE}}_{\rm{inc}} - {\rm{IE}}_{\rm{out}},$$

where IE_inc_ and IE_out_ are, respectively, the magnitudes of *C* exchanged at the incident and exit surfaces for the propagating light. In Fig. 2d are the numerical simulations of *C* for an unstructured flat Eu_2_O_3_ film in air. As expected, given the refractive indices (RI) of air (RI = 1) and polycarbonate (RI = 1.619) IE_inc_ > IE_out_ and ΔIE = 0.05 for both LCP and RCP light.

**Fig. 3:**
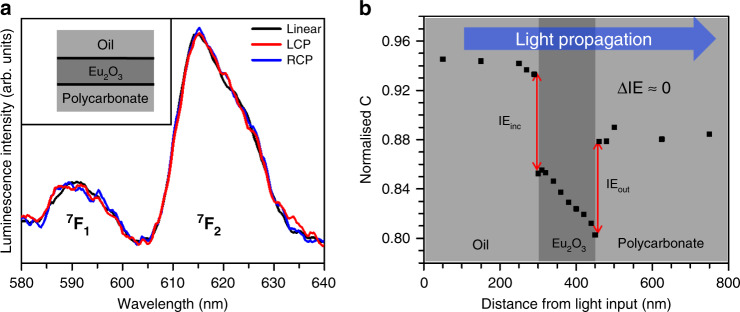
Optical chirality transfer with equivalent interfaces (oil-Eu_2_O_3_-polycarbonate). **a** Luminescence spectra normalised to ^7^F_1_ for LP (black) and LH (red) and RH (blue) CPL. **b** The equivalent numerical simulation to that is shown in Fig. 2d for the oil-Eu_2_O_3_- polycarbonate case, using thicknesses of 300 nm (oil), 150 nm (Eu_2_O_3_) and 350 nm (polycarbonate) for the three films.

By covering the air interface with microscope oil (RI = 1.518), the RI environment becomes closer to that of the polycarbonate substrate, so that the numerical simulations, Fig. 3a, give ΔIE ≈ 0 for both CP of light. In the absence of a net exchange of chirality, Fig. 3b shows no difference between LP and CP spectra, indicating no significant symmetry-reducing perturbation.

### Nanostructured Eu_2_O_3_ metafilms

**Fig. 4:**
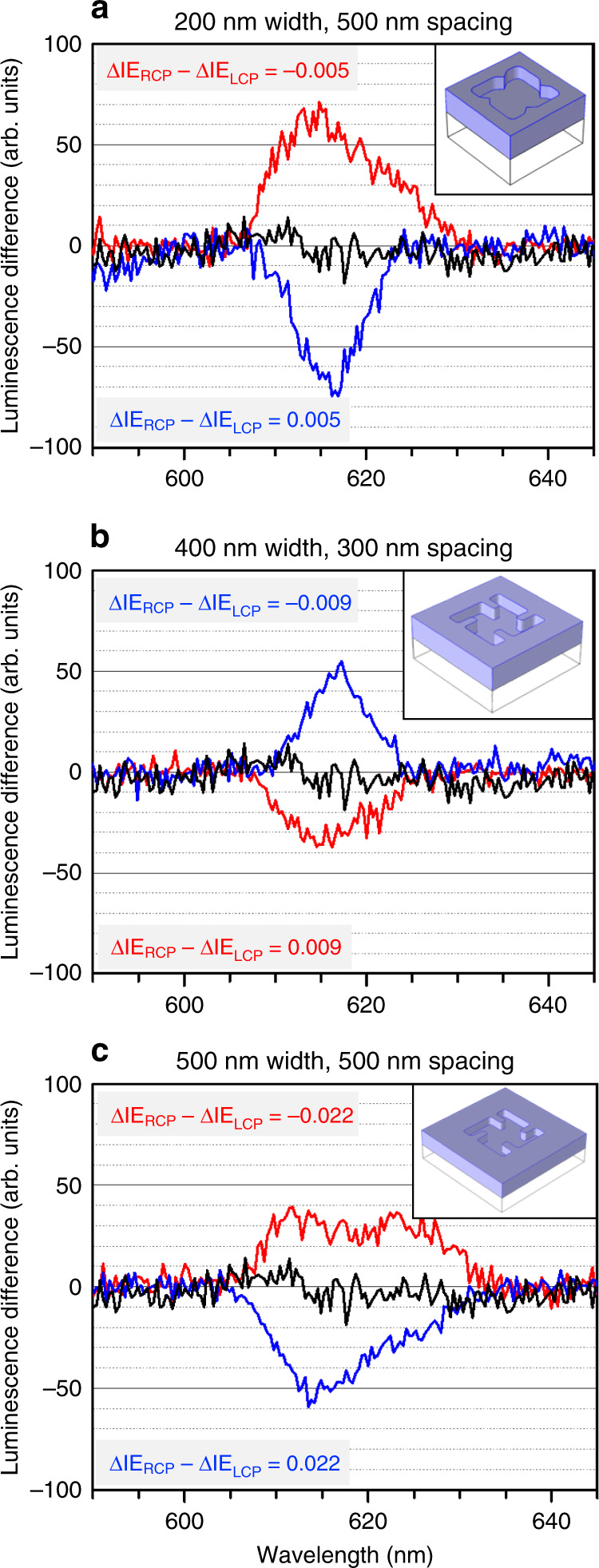
Luminescence difference spectra for metafilms. **a**, **c** show the difference in luminescence RCP–LCP for the LH nanostructure (red), RH nanostructure (blue) and equivalent unstructured surface (black). The LH and RH structures show opposite differences at the ^7^F_2_ peak. The width and spacing labels equate to the lateral size and interstitial spacing of the nanostructures, respectively, ΔIE_RCP_ − ΔIE_LCP_ values for each handedness of structure are shown (red and blue for LH and RH structures, respectively), demonstrating the same equal and opposite behaviour as the difference spectra. Insets show structure geometries not drawn to scale (see Supplementary Fig. 3 for scale diagrams).

In a second experiment, we modulated the magnitude of ΔIE using nanostructured chiral metafilms (see Supplementary Fig. 3 for images). The Eu_2_O_3_ from which these films are composed is identical to that above and is amorphous and achiral on atomic length scales, so that Eu^3+^ does not display any intrinsically chiral properties. Scattering CP and LP light from chiral nanostructures has previously been shown to create near fields, which exhibit spatial variations in both intensity and *C* (Supplementary Fig. 4). The values of *C* can locally be either greater or less than that of the incident CPL alone^30^. Consequently, the overall value of $( \pmb{\cal{E}} \times \pmb{\cal{E}}^\ast )$ in Eq. (2)—and hence the amount of *C* exchanged—can differ from that discussed in Figs. 2 and 3. The level of *C* transferred is dependent on the exact nature of the chiral structure. The metafilms used in this study were chosen because they do not give rise to final state effects that would affect the luminescence intensities (see Supplementary Note 2 and Supplementary Fig. 5). It should be noted that in contrast to unstructured surfaces, scattering of LP from chiral metafilms can also generate chiral near fields^31^ and thus transfer *C* to a film, albeit to a lesser extent than for CP light. Luminescence spectra were collected for LCP and RCP from left-handed (LH) and right-handed (RH) forms of each of the three types of chiral metafilms. To highlight the CP dependency, we present difference spectra (RCP minus LCP) for the three types of metamaterial, illustrated in Fig. 4 (see Supplementary Fig. 6 for individual spectra). As one would expect, the difference spectra collected from LH and RH structures are qualitatively equal and opposite. The signs of the difference spectra correlate with the difference in *C* transferred by RCP and LCP (ΔIE_RCP_ − ΔIE_LCP_). A detailed method for these calculations and the associated values are given in Supplementary Note 2 and Supplementary Table 1, respectively. A positive (negative) difference in the amount of *C* transferred corresponds to difference spectra with negative (positive) senses. The correlation between ΔIE_RCP_ − ΔIE_LCP_ and magnitude of the difference spectra is qualitative. A comparison between the asymmetry parameter, *g*, and the amount ΔIE_RCP_ − ΔIE_LCP_ is given in Supplementary Table 2. As expected, the 500 × 500 structure, which has the largest exchange of chirality, gives the largest *g*. It should be stressed that the idealised structures in the EM modelling procedure do not account for defects in the real structures, which may alter the amount of optical chirality transferred. There is no simple correlation between nanostructure geometry (i.e., width and spacing) and the amount of chirality transferred to the substrate. An absence of complete mirror symmetry within pairs of experimental difference spectra are a result of small variations in the geometries of the nanostructures that occur as a result of the fabrication process.

## Discussion

Extensive literature, since the 1960s, describes the use of Eu^3+^ luminescence to probe local co-ordination environment^25^, enabling the present data to be placed into context. The changes in ^7^F_2_ emission observed here are equivalent to those produced by ∼1% compressive lattice strain in crystalline systems, produced through ion substitution^32^ or the application of GPa pressures^33^. These levels of perturbation are significant and are sufficient, for example, to manipulate superconducting^34^ and magnetoresistive^35^ properties in other systems.

Creating meta-crystal fields through the exchange of optical chirality provides a mechanism to make a broad range of materials photo-responsive. Thus, the phenomenon lifts the constraints on materials that can be exploited in opto-electronics, thus providing the opportunity for novel functionalities. Given that the phenomenon originates from an interfacial mechanism it could be an ideal candidate for the optical control of topological properties.

## Methods

### Luminescence setup

The 404 nm laser diode was driven using a 180 mA fixed current, resulting in a maximum optical power output of ~17 mW measured using a digital power meter equipped with a photodiode sensor. Luminescence spectra in Figs. 2a, 3a and 4 were measured at this power. For power dependence measurements, shown in Fig. 2 and Supplementary Fig. 7, 17 mW was defined as 100% output. For excitation with LP light, luminescence spectra were collected using an orthogonal polarisation, which reduced the background. The same output polarisation was used to monitor luminescence excited by CPL. See Supplementary Fig. 8 for a diagram of the setup.

### Polycarbonate substrate fabrication

The templated substrates were made using an injection moulding machine (ENGEL), following the technique described by Gadegaard et al.^36^. The master shim for this was made using electron beam lithography. To create the master, 100 nm of poly(methyl methacrylate) resist was spin coated onto a Si wafer and baked for an hour at 180 °C. The resist is patterned using a VB6 UHR EWF lithography tool (Raith). The exposed resist was developed in IPA and methyl isobutyl ketone, MIBK (3:1 ratio) for 60 s, then rinsed in copious amounts of IPA before dried in a stream of nitrogen gas. The shim for injection moulding was prepared by electroplating from the patterned resist master. The nano patterns were indentations in the surface and had a depth of ~80 nm, range from 200 to 500 nm in length from arm to arm, and inter-structure spacing from 300 to 500 nm. When Eu_2_O_3_ was deposited on the surface, it uniformly coats the substrate and takes the shape of the indentation to form a structure constituting an “inverse” hole structure at the top and a solid one at the bottom. The nanostructures have subsequently been given labels of the general form (*A* × *B*), where *A* and *B* are, respectively, the lateral dimensions and separations of nanostructures in nanometres.

### Eu_2_O_3_ deposition

Films were deposited by pulsed laser deposition (PLD) using sintered Eu_2_O_3_ targets (Pi Kem Ltd., UK) in 50mTorr pressure of oxygen. Deposition was conducted at room temperature directly onto structured polycarbonate substrates in a Neocera Pioneer PLD vacuum system, employing a Coherent Compex Pro KrF excimer laser (248 nm, 20 ns pulses, 18 Hz, up to 200 mJ pulse energy). The target was rotated during deposition to minimise the transfer of particulates to the sample. The film structure was then characterised by transmission electron microscopy by deposition a ~40-nm-thick film directly onto an amorphous holey carbon film and imaged in a JEOL ARM CFEG instrument operated at 200 kV. Supplementary Fig. 2 presents a typical image of the edge of the film, which appears structureless. Neither a Fourier transform nor a selected area electron diffraction image (both inset to the figure) reveals sharp spots or rings that would indicate crystallinity. A single, diffuse ring is observed in diffraction that is characteristic of an amorphous, or glassy material.

### Numerical electromagnetic modelling

EM simulations were performed using a commercial finite-element package (COMSOL v4.4, Wave optics module). Periodic boundary conditions were used to emulate the meta-film arrays. Perfectly matched layer conditions were used above and below the input and output ports. Varying polarisations of EM wave were applied at normal incidence onto the films. COMSOL uses the finite-element method to solve Maxwell’s equations over a specified geometry with fields and optical chirality being measured at pre-defined surfaces above, within, and below the films. For Eu_2_O_3_ interpolation functions in terms of wavelength for the real and imaginary parts of refractive index were identified from two sources^37,38^ and each implemented to identify a best fit compared to experimental data. Paramagnetic behaviour of the Eu_2_O_3_ was accounted for by including a relative magnetic permeability of 1.01^38^.

## Supplementary information

Supplementary Information

## Data Availability

Raw data that support the findings of this study are available from the author upon request, and are available in Zenodo with the identifier 10.5281/zenodo.3939181.

## References

[1] Wang Q (2016). Optically reconfigurable metasurfaces and photonic devices based on phase change materials. Nat. Photonics.

[2] Nova TF, Disa AS, Fechner M, Cavalleri A (2019). Metastable ferroelectricity in optically strained SrTiO_3_. Science.

[3] Li X (2019). Terahertz field-induced ferroelectricity in quantum paraelectric SrTiO_3_. Science.

[4] Fausti D (2011). Light-Induced superconductivity in a stripe-ordered cuprate. Science.

[5] Zong A (2019). Evidence for topological defects in a photoinduced phase transition. Nat. Phys..

[6] Kogar A (2020). Light-induced charge density wave in LaTe_3_. Nat. Phys..

[7] Sie EJ (2019). An ultrafast symmetry switch in a Weyl semimetal. Nature.

[8] Shimomura K, Ikai T, Kanoh S, Yashima E, Maeda K (2014). Switchable enantioseparation based on macromolecular memory of a helical polyacetylene in the solid state. Nat. Chem..

[9] Noorduin WL (2009). Complete chiral symmetry breaking of an amino acid derivative directed by circularly polarized light. Nat. Chem..

[10] Wu S-T (2014). Enantioselective synthesis of a chiral coordination polymer with circularly polarized visible laser. Angew. Chem. Int. Ed..

[11] Kim MJ, Shin BG, Kim JJ, Kim DY (2002). Photoinduced supramolecular chirality in amorphous azobenzene polymer films. J. Am. Chem. Soc..

[12] Ashkin A (1980). Applications of laser-radiation pressure. Science.

[13] Babiker M, Power WL, Allen L (1994). Light-induced torque on moving atoms. Phys. Rev. Lett..

[14] Padgett M, Bowman R (2011). Tweezers with a twist. Nat. Photonics.

[15] Juan ML, Righini M, Quidant R (2011). Plasmon nano-optical tweezers. Nat. Photonics.

[16] Lipkin DM (1964). Existence of new conservation law in electromagnetic theory. J. Math. Phys..

[17] Proskurin, I., Ovchinnikov, A. S., Nosov, P. & Kishine, J. Optical chirality in gyrotropic media: symmetry approach. *New. J. Phys.* **19**10.1088/1367-2630/aa6acd (2017).

[18] Tang YQ, Cohen AE (2011). Enhanced enantioselectivity in excitation of chiral molecules by superchiral light. Science.

[19] Bliokh, K. Y. & Nori, F. Characterizing optical chirality. *Phys. Rev. A* **83**10.1103/PhysRevA.83.021803 (2011).

[20] Poulikakos LV (2016). Optical chirality flux as a useful far-field probe of chiral near fields. ACS Photonics.

[21] Cameron RP, Barnett SM, Yao AM (2012). Optical helicity, optical spin and related quantities in electromagnetic theory. New. J. Phys..

[22] Barnett, S. M., Cameron, R. P. & Yao, A. M. Duplex symmetry and its relation to the conservation of optical helicity. *Phys. Rev. A* **86**10.1103/PhysRevA.86.013845 (2012).

[23] van Kruining K, Götte JB (2016). The conditions for the preservation of duality symmetry in a linear medium. J. Opt..

[24] Van Vleck JH (1932). Theory of the variations in paramagnetic anisotropy among different salts of the iron group. Phys. Rev..

[25] Binnemans K (2015). Interpretation of europium(III) spectra. Coord. Chem. Rev..

[26] Mariscal A (2016). Tuning Eu3+ emission in europium sesquioxide films by changing the crystalline phase. Appl. Surf. Sci..

[27] Sheng KC, Korenowski GM (1988). Laser-induced optical-emission studies of Eu-3+ sites in polycrystalline powders of monoclinic and body-centered cubic Eu2o3. J. Phys. Chem..

[28] Yakel HL (1979). Refinement of the crystal-structure of monoclinic europium sesquioxide. Acta Crystallogr. B.

[29] Tanner PA (2013). Some misconceptions concerning the electronic spectra of tri-positive europium and cerium. Chem. Soc. Rev..

[30] Hendry E (2010). Ultrasensitive detection and characterization of biomolecules using superchiral fields. Nat. Nanotechnol..

[31] Kelly C (2018). Chiral plasmonic fields probe structural order of biointerfaces. J. Am. Chem. Soc..

[32] Villafuerte-Castrejon ME (2007). Luminescence and structural study of Bi4-xEuxTi3O12 solid solution. J. Eur. Ceram. Soc..

[33] Zhang, J. et al. Photoluminescence studies of Y2O3:Eu3+ under high pressure. *J. Appl. Phys*. **115**10.1063/1.4861386 (2014).

[34] Kimber SAJ (2009). Similarities between structural distortions under pressure and chemical doping in superconducting BaFe2As2. Nat. Mater..

[35] Manna, R. S. et al. Lattice strain accompanying the colossal magnetoresistance effect in EuB6. *Phys. Rev. Lett.* **113**10.1103/PhysRevLett.113.067202 (2014). 10.1103/PhysRevLett.113.067202 25148347

[36] Gadegaard N, Mosler S, Larsen NB (2003). Biomimetic polymer nanostructures by injection molding. Macromol. Mater. Eng..

[37] Jayaraj MK, Vallabhan CPG (1989). Dielectric and optical-properties of europium oxide-films. Thin Solid Films.

[38] Weast, R. C. (ed.). *CRC Handbook of Chemistry and Physics,* 1st student edn (Pagination Varies CRC Press, Inc., Boca Raton, 1988).

