# Supplementary material for: Controlling the symmetry of inorganic ionic nanofilms with optical chirality
Source: Nat Commun. 2020 Oct 14;11:5169. doi: 10.1038/s41467-020-18869-9 (PMC7560753; doi:10.1038/s41467-020-18869-9)
Supplement: Supplementary file 1 — Supplementary Information [file 41467_2020_18869_MOESM1_ESM.pdf]

Supplementary Information

Controlling the symmetry of inorganic ionic nanofilms with optical chirality

Christopher Kelly[1*], Donald A. Maclaren[2], Katie McKay[1], Anthony McFarlane[1], Affar Karimullah[1], Nikolaj Gadegaard[3], Laurence D. Barron[1], Sonja Franke-Arnold[2], Frances Crimin[2] Jörg B. Götte[2,4], Stephen M. Barnett[2] and Malcolm Kadodwala[1*].

Correspondence to: malcolm.kadodwala@glasgow.ac.uk, c.kelly.4@research.gla.ac.uk.

Supplementary Note 1 – Derivation of Chiral Continuity Equation

In the following we present a derivation of the continuity equation for the optical chirality in the presence of a medium. In contrast to Poulikakos *et al1*[1]. we do not require the chirality to be split into electric and magnetic parts, but work instead with the complex electric permittivity and magnetic permeability directly. We assume the material properties vary little over a narrow band of frequencies, but they are otherwise fully general.

The optical chirality density in a medium as described in the main text is given by

$$C = \frac{1}{2}\left(\boldsymbol{B}\cdot\dot{\boldsymbol{D}} - \boldsymbol{D}\cdot\dot{\boldsymbol{B}}\right), \qquad \text{[S1]}$$

where $\boldsymbol{D}$ is the electric displacement field, $\boldsymbol{B}$ is magnetic flux density, and $\dot{\boldsymbol{D}}$ and $\dot{\boldsymbol{B}}$ are their respective time derivatives. Differentiating w.r.t. time and using Maxwell's equations (in the absence of charges and currents) gives

$$\dot{C} = \frac{1}{2}\left(\boldsymbol{B}\cdot\nabla\times\dot{\boldsymbol{H}} + \boldsymbol{D}\cdot\nabla\times\dot{\boldsymbol{E}}\right), \qquad \text{[S2]}$$

In order to construct a continuity equation, note the identities in equations S3, and S4

$$\nabla\cdot\left(\boldsymbol{D}\times\dot{\boldsymbol{E}}\right) = \dot{\boldsymbol{E}}\cdot(\boldsymbol{\nabla}\times\boldsymbol{D}) - \boldsymbol{D}\cdot\left(\boldsymbol{\nabla}\times\dot{\boldsymbol{E}}\right), \qquad \text{[S3]}$$

$$\nabla\cdot\left(\boldsymbol{B}\times\dot{\boldsymbol{H}}\right) = \dot{\boldsymbol{H}}\cdot(\boldsymbol{\nabla}\times\boldsymbol{B}) - \boldsymbol{B}\cdot\left(\boldsymbol{\nabla}\times\dot{\boldsymbol{H}}\right), \qquad \text{[S4]}$$

recognising the third term in each of these from equation S2.

Thus a continuity equation can be constructed using equations S2, S3 and S4

$$\dot{C} - \frac{1}{2}\nabla \cdot \left[\dot{\boldsymbol{E}} \times \boldsymbol{D} + \dot{\boldsymbol{H}} \times \boldsymbol{B}\right] = \frac{1}{2}\left[\dot{\boldsymbol{E}} \cdot (\boldsymbol{\nabla} \times \boldsymbol{D}) + \dot{\boldsymbol{H}} \cdot (\boldsymbol{\nabla} \times \boldsymbol{B})\right], \quad \text{[S5]}$$

in which we assign the second term on the left side as chirality flux, and the right-hand side as the chirality source/sink. This now requires justification.

First a complex permittivity $\varepsilon = \varepsilon' + i\varepsilon''$ and permeability $\mu = \mu' + i\mu''$ are introduced and assuming that the incident light is sufficiently narrow band, ε and μ can be approximated as being constant. In addition, the four fields of interest can be written as

$$E = (\boldsymbol{\mathcal{E}} + \boldsymbol{\mathcal{E}}^*) \quad \text{[S6]}$$
$$\boldsymbol{H} = (\boldsymbol{\mathcal{H}} + \boldsymbol{\mathcal{H}}^*) \quad \text{[S7]}$$
$$\boldsymbol{D} = (\varepsilon\, \boldsymbol{\mathcal{E}} + \varepsilon^* \boldsymbol{\mathcal{E}}^*) \quad \text{[S8]}$$
$$B = (\mu \boldsymbol{\mathcal{H}} + \mu^* \boldsymbol{\mathcal{H}}^*) \quad \text{[S9]}$$

The flux term (ignoring the divergence for now) in equation S5 then becomes S10 and with the narrow-band light assumption (i.e. Δω << ω), the approximation that, for example $\dot{\boldsymbol{\mathcal{E}}} = -i\omega\boldsymbol{\mathcal{E}}$**,** and so on which gives S11

$$\frac{1}{2}\left[\dot{\boldsymbol{E}} \times \boldsymbol{D} + \dot{\boldsymbol{H}} \times \boldsymbol{B}\right] = \frac{1}{2}\left[\left(\dot{\boldsymbol{\mathcal{E}}} + \dot{\boldsymbol{\mathcal{E}}}^*\right) \times (\varepsilon\, \boldsymbol{\mathcal{E}} + \varepsilon^* \boldsymbol{\mathcal{E}}^*) + \left(\dot{\boldsymbol{\mathcal{H}}} + \dot{\boldsymbol{\mathcal{H}}}^*\right) \times (\mu \boldsymbol{\mathcal{H}} + \mu^* \boldsymbol{\mathcal{H}}^*)\right], \quad \text{[S10]}$$
$$= \frac{1}{2}[(-i\omega\boldsymbol{\mathcal{E}} + i\omega\boldsymbol{\mathcal{E}}^*) \times (\varepsilon\, \boldsymbol{\mathcal{E}} + \varepsilon^* \boldsymbol{\mathcal{E}}^*) + (-i\omega\boldsymbol{\mathcal{H}} + i\omega\boldsymbol{\mathcal{H}}^*) \times (\mu \boldsymbol{\mathcal{H}} + \mu^* \boldsymbol{\mathcal{H}}^*)],$$
$$= \frac{1}{2}[-i\omega\varepsilon^* \boldsymbol{\mathcal{E}} \times \boldsymbol{\mathcal{E}}^* - i\omega\varepsilon \boldsymbol{\mathcal{E}} \times \boldsymbol{\mathcal{E}}^* - i\omega\mu^* \boldsymbol{\mathcal{H}} \times \boldsymbol{\mathcal{H}}^* - i\omega\mu \boldsymbol{\mathcal{H}} \times \boldsymbol{\mathcal{H}}^*],$$
$$= \frac{-i\omega}{2}[(\varepsilon^* + \varepsilon)\boldsymbol{\mathcal{E}} \times \boldsymbol{\mathcal{E}}^* + (\mu^* + \mu)\boldsymbol{\mathcal{H}} \times \boldsymbol{\mathcal{H}}^*],$$
$$= -i\omega[\varepsilon'(\boldsymbol{\mathcal{E}} \times \boldsymbol{\mathcal{E}}^*) + \mu'(\boldsymbol{\mathcal{H}} \times \boldsymbol{\mathcal{H}}^*)], \quad \text{[S11]}$$

This works as a flux as only the real parts of the permittivity and permeability appear, there is no connection to losses and these terms are unchanged under the limit of zero loss.

Now, the source/sink term in equation S5 can be rewritten, as with the flux, as

$$\frac{1}{2}[(-i\omega\boldsymbol{\mathcal{E}} + i\omega\boldsymbol{\mathcal{E}}^*) \cdot \boldsymbol{\nabla} \times (\varepsilon\, \boldsymbol{\mathcal{E}} + \varepsilon^* \boldsymbol{\mathcal{E}}^*) + (-i\omega\boldsymbol{\mathcal{H}} + i\omega\boldsymbol{\mathcal{H}}^*) \cdot \boldsymbol{\nabla} \times (\mu \boldsymbol{\mathcal{H}} + \mu^* \boldsymbol{\mathcal{H}}^*)] \quad \text{[S12]}$$
$$= \frac{1}{2}[(-i\omega\boldsymbol{\mathcal{E}} + i\omega\boldsymbol{\mathcal{E}}^*) \cdot (\varepsilon\, \boldsymbol{\nabla} \times \boldsymbol{\mathcal{E}} - \boldsymbol{\mathcal{E}} \times \boldsymbol{\nabla}\varepsilon + \varepsilon^* \boldsymbol{\nabla} \times \boldsymbol{\mathcal{E}}^* - \boldsymbol{\mathcal{E}}^* \times \boldsymbol{\nabla}\varepsilon^*)$$
$$+ (-i\omega\boldsymbol{\mathcal{H}} + i\omega\boldsymbol{\mathcal{H}}^*) \cdot (\mu \boldsymbol{\nabla} \times \boldsymbol{\mathcal{H}} - \boldsymbol{\mathcal{H}} \times \boldsymbol{\nabla}\mu + \mu^* \boldsymbol{\nabla} \times \boldsymbol{\mathcal{H}}^* - \boldsymbol{\mathcal{H}}^* \times \boldsymbol{\nabla}\mu^*)] \quad \text{[S13]}$$

Before continuing, note that $\nabla \times \boldsymbol{\mathcal{E}} = i\omega\boldsymbol{\mathcal{B}} = i\omega\mu\boldsymbol{\mathcal{H}}$ and $\nabla \times \boldsymbol{\mathcal{H}} = -i\omega\boldsymbol{\mathcal{D}} = -i\omega\varepsilon\boldsymbol{\mathcal{E}}$**.** First taking only the terms containing the curls of $\boldsymbol{\mathcal{E}}$ and $\boldsymbol{\mathcal{H}}$ and their complex conjugates,

$$(-i\omega\boldsymbol{\mathcal{E}} + i\omega\boldsymbol{\mathcal{E}}^*) \cdot (\varepsilon\, \boldsymbol{\nabla} \times \boldsymbol{\mathcal{E}} + \varepsilon^* \boldsymbol{\nabla} \times \boldsymbol{\mathcal{E}}^*) + (-i\omega\boldsymbol{\mathcal{H}} + i\omega\boldsymbol{\mathcal{H}}^*) \cdot (\mu \boldsymbol{\nabla} \times \boldsymbol{\mathcal{H}} + \mu^* \boldsymbol{\nabla} \times \boldsymbol{\mathcal{H}}^*)$$

$$= (-i\omega\boldsymbol{\mathcal{E}} + i\omega\boldsymbol{\mathcal{E}}^*) \cdot (i\omega\varepsilon\mu\boldsymbol{\mathcal{H}} - i\omega\varepsilon^*\mu^*\boldsymbol{\mathcal{H}}^*) + (-i\omega\boldsymbol{\mathcal{H}} + i\omega\boldsymbol{\mathcal{H}}^*) \cdot (-i\omega\varepsilon\mu\boldsymbol{\mathcal{E}} + i\omega\varepsilon^*\mu^*\boldsymbol{\mathcal{E}}^*)$$

$$= \omega^2(\varepsilon\mu - \varepsilon^*\mu^*)(\boldsymbol{\mathcal{E}} \cdot \boldsymbol{\mathcal{H}}^* - \boldsymbol{\mathcal{E}}^* \cdot \boldsymbol{\mathcal{H}})$$

$$= 2i\omega^2(\varepsilon'\mu'' + \varepsilon''\mu')(\boldsymbol{\mathcal{E}} \cdot \boldsymbol{\mathcal{H}}^* - \boldsymbol{\mathcal{E}}^* \cdot \boldsymbol{\mathcal{H}}) \quad \text{[S14]}$$

Now, taking the remaining terms from equation S13, and simplifying using the scalar triple product gives

$$(-i\omega\boldsymbol{\mathcal{E}} + i\omega\boldsymbol{\mathcal{E}}^*) \cdot (-\boldsymbol{\mathcal{E}} \times \boldsymbol{\nabla}\varepsilon - \boldsymbol{\mathcal{E}}^* \times \boldsymbol{\nabla}\varepsilon^*) + (-i\omega\boldsymbol{\mathcal{H}} + i\omega\boldsymbol{\mathcal{H}}^*) \cdot (-\boldsymbol{\mathcal{H}} \times \boldsymbol{\nabla}\mu - \boldsymbol{\mathcal{H}}^* \times \boldsymbol{\nabla}\mu^*)$$

$$= +i\omega(\boldsymbol{\mathcal{E}} \times \boldsymbol{\mathcal{E}}^*) \cdot \boldsymbol{\nabla}\varepsilon^* - i\omega(\boldsymbol{\mathcal{E}}^* \times \boldsymbol{\mathcal{E}}) \cdot \boldsymbol{\nabla}\varepsilon + i\omega(\boldsymbol{\mathcal{H}} \times \boldsymbol{\mathcal{H}}^*) \cdot \boldsymbol{\nabla}\mu^* - i\omega(\boldsymbol{\mathcal{H}}^* \times \boldsymbol{\mathcal{H}}) \cdot \boldsymbol{\nabla}\mu$$

$$= +i\omega(\boldsymbol{\mathcal{E}} \times \boldsymbol{\mathcal{E}}^*) \cdot \boldsymbol{\nabla}(\varepsilon^* + \varepsilon) + i\omega(\boldsymbol{\mathcal{H}} \times \boldsymbol{\mathcal{H}}^*) \cdot \boldsymbol{\nabla}(\mu^* + \mu)$$

$$= i2\omega[\nabla\varepsilon' \cdot (\boldsymbol{\mathcal{E}} \times \boldsymbol{\mathcal{E}}^*) + \nabla\mu' \cdot (\boldsymbol{\mathcal{H}} \times \boldsymbol{\mathcal{H}}^*)] \qquad \text{[S15]}$$

Equation S15 involves only the gradients of the real parts of the permittivity and permeability but, given helicity and hence chirality change sign on reflection from an interface, the surface can act as a source of chirality even if the permittivity and permeability are real.

Thus, the source/sink term can be fully written as

$$i\omega^2(\boldsymbol{\mathcal{E}} \cdot \boldsymbol{\mathcal{H}}^* - \boldsymbol{\mathcal{E}}^* \cdot \boldsymbol{\mathcal{H}})(\mu'\varepsilon'' - \mu''\varepsilon') + i\omega[\nabla\varepsilon' \cdot (\boldsymbol{\mathcal{E}} \times \boldsymbol{\mathcal{E}}^*) + \nabla\mu' \cdot (\boldsymbol{\mathcal{H}} \times \boldsymbol{\mathcal{H}}^*)] \qquad \text{[S16]}$$

Therefore, by pulling equations S5, S11 and S16 together, the narrow bandwidth continuity equation can be written as

$$\dot{C} - i\omega\nabla \cdot \underbrace{[\varepsilon'(\boldsymbol{\mathcal{E}} \times \boldsymbol{\mathcal{E}}^*) + \mu'(\boldsymbol{\mathcal{H}} \times \boldsymbol{\mathcal{H}}^*)]}_{\text{chirality flux}} = +i\omega^2 \underbrace{(\boldsymbol{\mathcal{E}} \cdot \boldsymbol{\mathcal{H}}^* - \boldsymbol{\mathcal{H}} \cdot \boldsymbol{\mathcal{E}}^*)(\mu'\varepsilon'' + \varepsilon'\mu'')}_{\text{loss term}} +$$

$$i\omega \underbrace{[(\nabla\varepsilon') \cdot (\boldsymbol{\mathcal{E}} \times \boldsymbol{\mathcal{E}}^*) + (\nabla\mu') \cdot (\boldsymbol{\mathcal{H}} \times \boldsymbol{\mathcal{H}}^*)]}_{\text{interface−exchange term } (IE)},$$

which is equation (2) in the main text. In this form the continuity equation agrees with our earlier work for chiral media[2].

Supplementary Note 2 – Characterisation of Eu_2O_3 films

The optical properties of the meta-films were characterised using reflectance and optical rotation dispersion spectra (ORD). The meta-films used in this study were chosen because they displayed optical properties which would minimise the potential of final state effects influencing the luminescence properties. Firstly, structures chosen did not exhibit reflectance resonances which overlapped with the luminescence spectra. This for instance prevents enhanced emission induced by the Purcell effect. In Supplementary Figure 5(a) are reflectance spectra (relative to an unstructured flat surface) for the three meta-films used. In addition, for comparison, a spectrum from a metafilm (200 x 200) which displays significant resonances in the luminescence region is shown. To further limit the influence of final state effects an additional criterion, the absence of measurable optical rotation within the luminescence range, was also used. In Supplementary Figure 5(b-d) are ORD spectra showing no measurable optical rotation within the luminescence wavelength range.

Supplementary Note 3 - *C* calculated with COMSOL.

Supplementary Table 1 gives the *C* values calculated at each integration surface in the simulations. The CPL light input into the simulation has a *C* value which is normalised to 1. However, when incident light is reflected from the Eu_2O_3 film the sign of *C* is reversed (*i.e.* it becomes negative mimicking the reversal of the handedness of CPL on reflection). Consequently, due to the combining of the incident and reflected light, $C > 1$ in region between the input port of the model and the Eu_2O_3 surface. The values of *C* in this region and that of the light transmitted through the film are referred to as *C* incidence and *C* transmission respectively. The difference between *C* incidence and *C* transmission is the sum of the net exchange of *C* with the film and the amount of *C* dissipated by optical absorption (*C*(Exchanged + Dissipated)). Given that Eu_2O_3 is achiral, the dissipation term is identical for both LCP and RCP. Consequently, the difference between *C*(Exchange + dissipation) values obtained for LCP and RCP would be the differential amount of net *C* exchanged, which are the ΔIE_{RCP} -ΔIE_{LCP} values quoted in Figure 4 of the main text.

Supplementary References

1 Poulikakos, L. V. *et al.* Optical Chirality Flux as a Useful Far-Field Probe of Chiral Near Fields. *Acs Photonics* **3**, 1619-1625, doi:10.1021/acsphotonics.6b00201 (2016).

2 Crimin, F., Mackinnon, N., Gotte, J. B. & Barnett, S. M. On the conservation of helicity in a chiral medium. *Journal of Optics* **21**, 6, doi:10.1088/2040-8986/ab387c (2019).

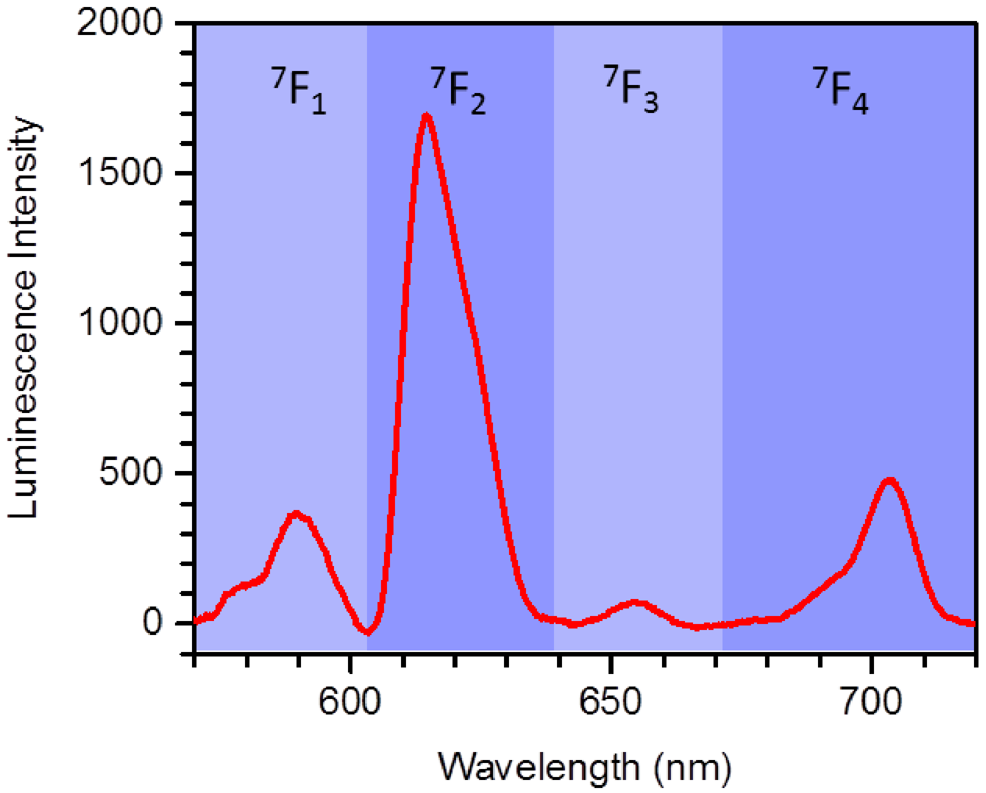

Supplementary Figure 1. Luminescence spectrum of unstructured Eu_2O_3 film. This shows the four $^5D_0 \rightarrow {}^7F_J$ transition luminescence peaks that are observed in the experiments, noting that the $^7F_2$ peak is hypersensitive and so is the focus in the main text. The example given here is for the flat (non-nanostructured) film, illuminated with linearly polarised light.

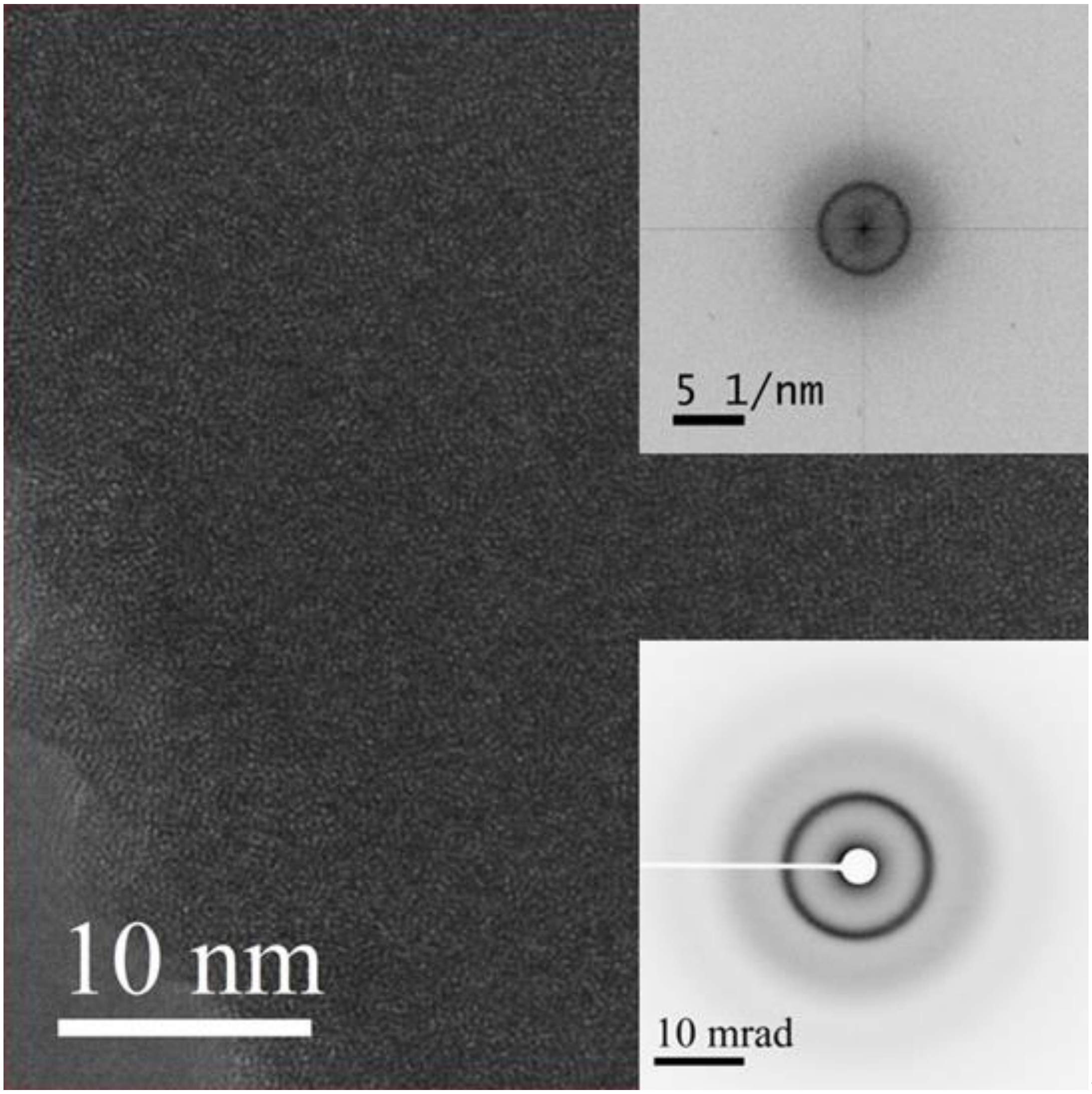

<

Supplementary Figure 2. Transmission electron microscopy of a Eu_2O_3 film. The deposited film lacks the interference fringes that are characteristic of a crystalline material. A Fourier transform (inset, top) and a selected electron diffraction pattern (inset, bottom) similarly lack sharp diffractive features; the film is amorphous. The amorphous nature agrees with a previous PLD study, where it was also shown that the chemical nature of films grown under similar conditions was predominantly Eu_2O_3. That study also noted a small fraction of EuO, attributed to chemical reduction during laser ablation, which is here expected to be minimised by the depositing in a low-pressure oxygen gas background.

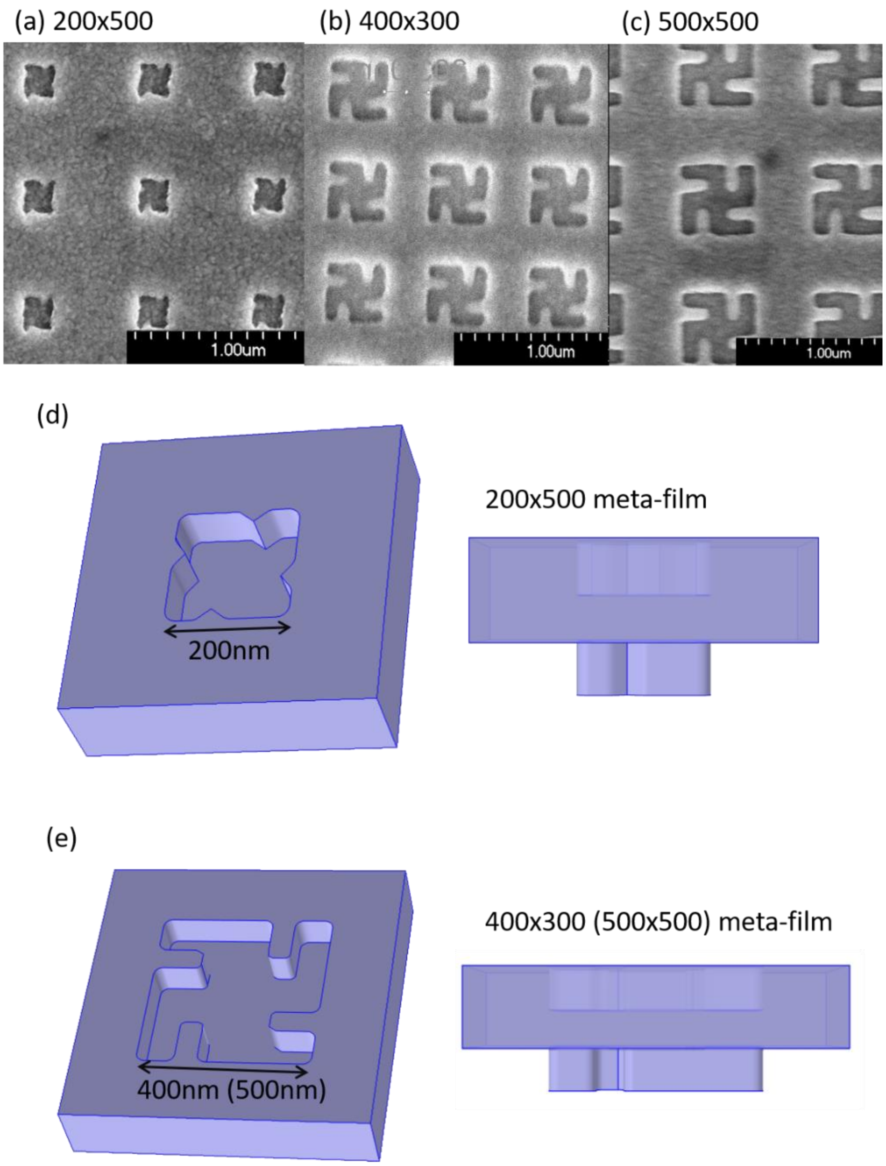

Supplementary Figure 3. Images of the nanostructures used in experiment and simulation. Scanning electron microscope images of the three meta-films used in experiment are shown in (a-c), with 1 µm scalebars at the bottom right. Idealised models of the structures used for electromagnetic modelling of the three films from a perspective (left) view and cross-section (right) view, showing the solid structure with the inverse structure above it, are in (d-e).

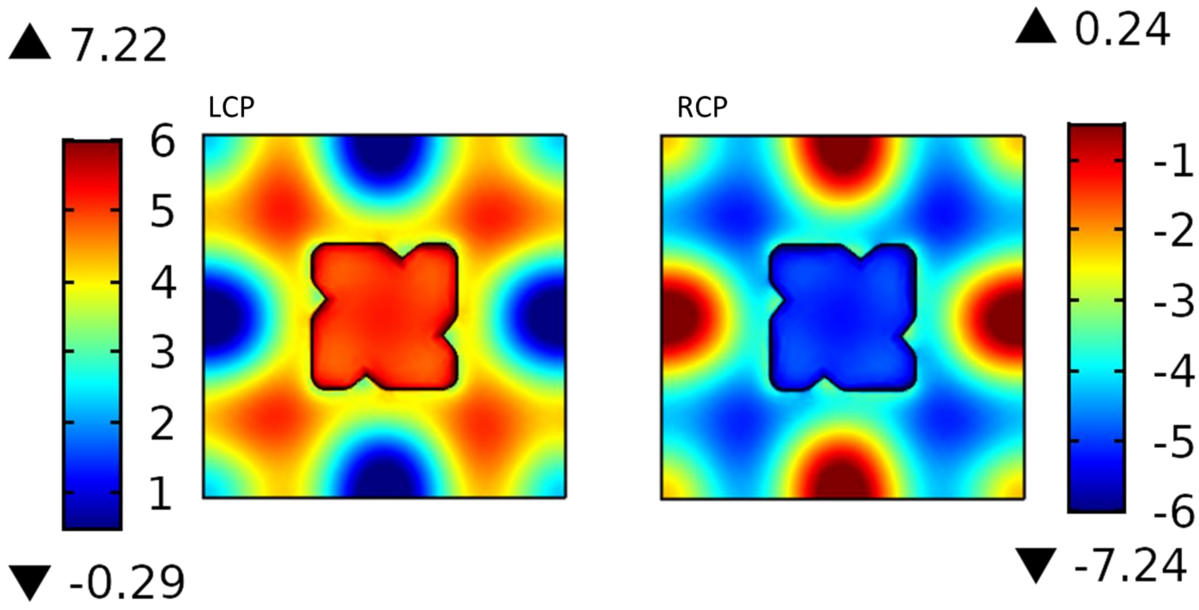

Supplementary Figure 4. Colormap showing spatial distribution of *C* for the 200x500 meta-film. From the numerical simulations, *C* can be calculated at each surface, such that it can be plotted to give a visualisation of its spatial distribution, as illuminated by LCP (left image) and RCP (right image), as well as integrated over that surface in order to calculate the ΔIE values. Dark red represents maximum positive values and dark blue highest magnitude negative values, given on the scale bars, and are relative to circularly polarised light in air. In this case *C* has been normalized to LCP.

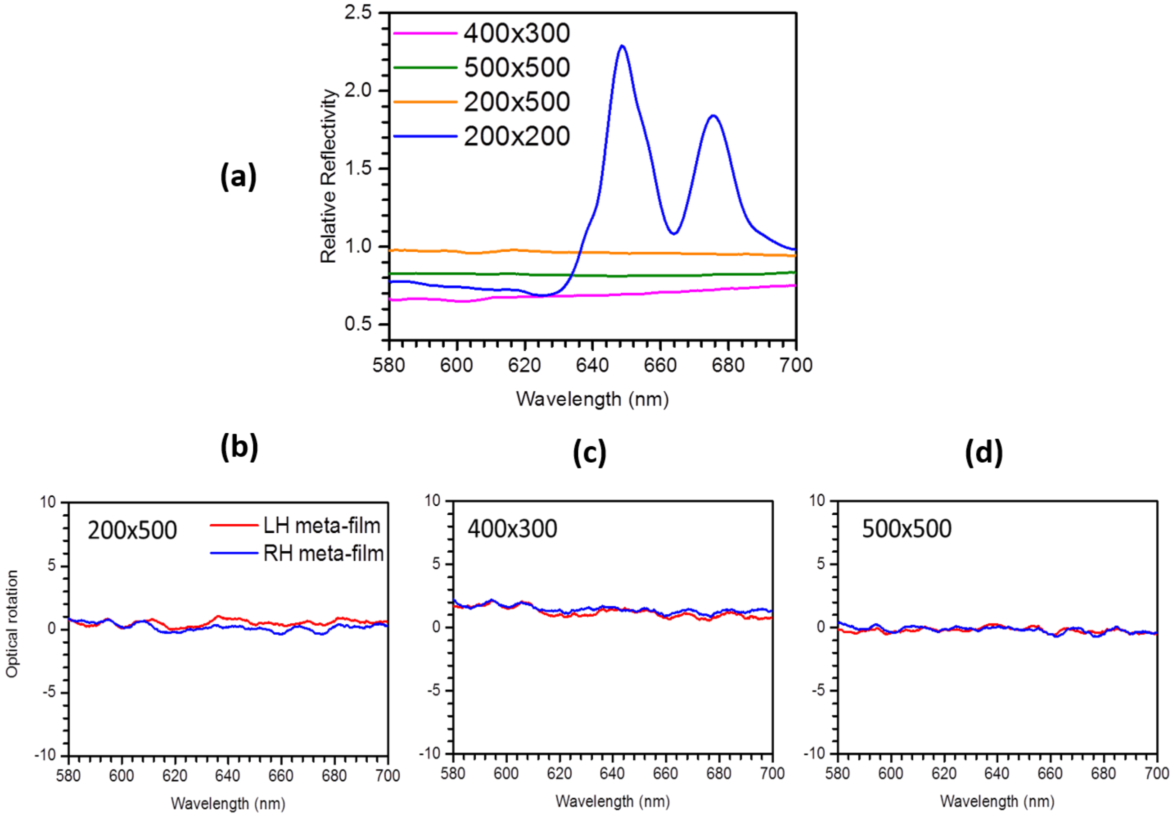

Supplementary Figure 5. Characterisation of Eu_2O_3 films. (a) Reflectance spectra for those meta-films used in the main text but with the addition of a structure which exhibits resonances, for comparison. (b, c, d) ORD spectra of meta-films used in the experiments. See Supplementary Note 2 for more details.

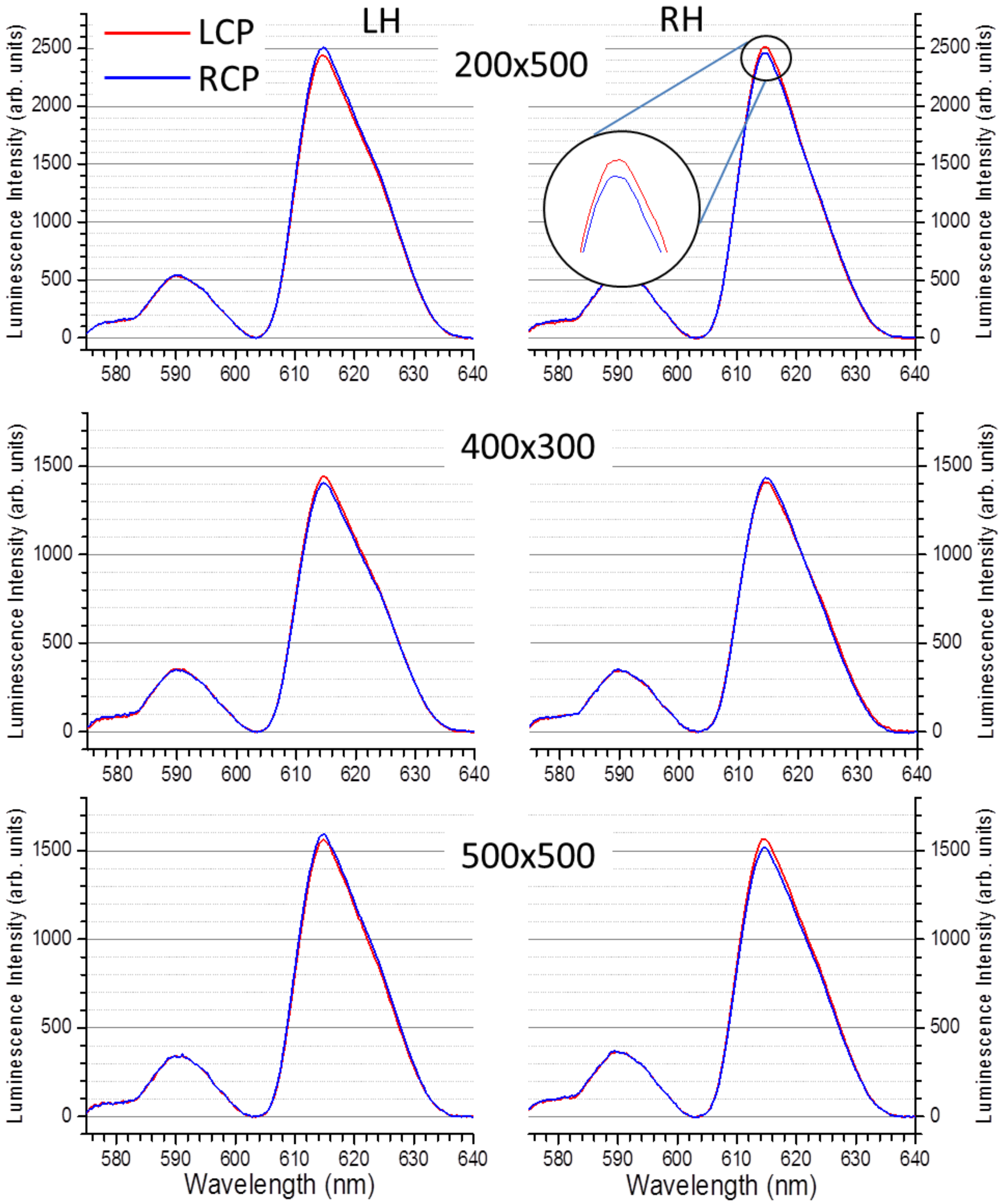

Supplementary Figure 6. Luminescence spectra for meta-film excited by CPL. Luminescence spectra are shown for the 200x500, 400x300 and 500x500 metafilms, including the LH (left side) and RH (right side) structures. The films were measured being illuminated with LCP (red) and RCP (blue) light. The inset in the top panel is a close-up of the change in $^7F_2$ transition peak from LCP to RCP, seen for each meta-film.

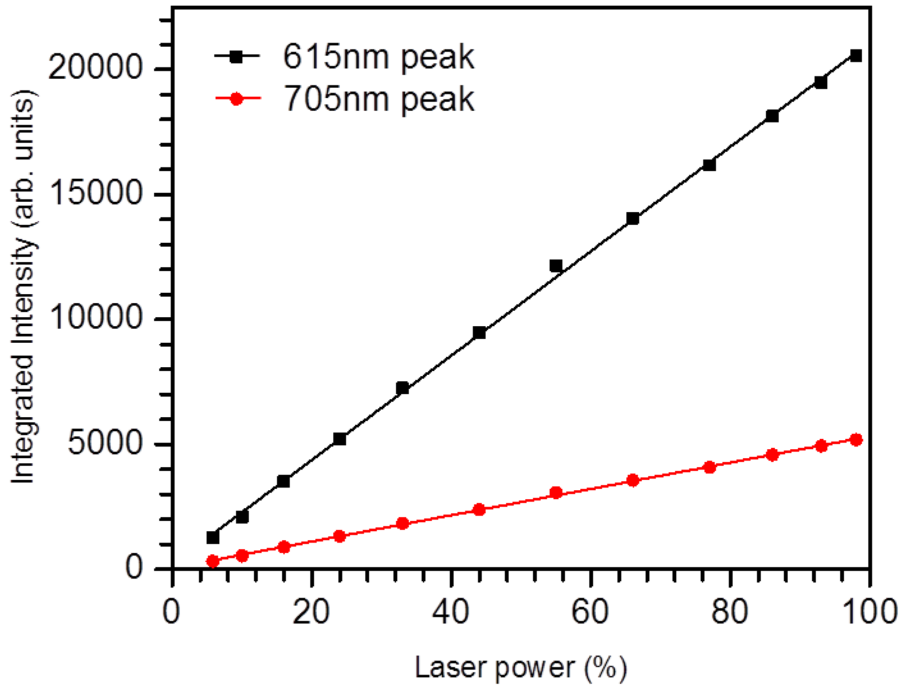

Supplementary Figure 7. Power dependence of luminescence intensity for the unstructured Eu_2O_3 film. The sample used for this measurement was illuminated with linearly polarised light. The power dependencies of the flat surface and structures are calculated by dividing the integrated $^7F_2$ (615nm) peak by the integrated $^7F_4$ (705nm) peak (100% laser power ~17mW), to demonstrate relative changes with power for the meta-film. This shows those integrated values before being divided for the flat surface, to show that there is no saturation of the emission signal.

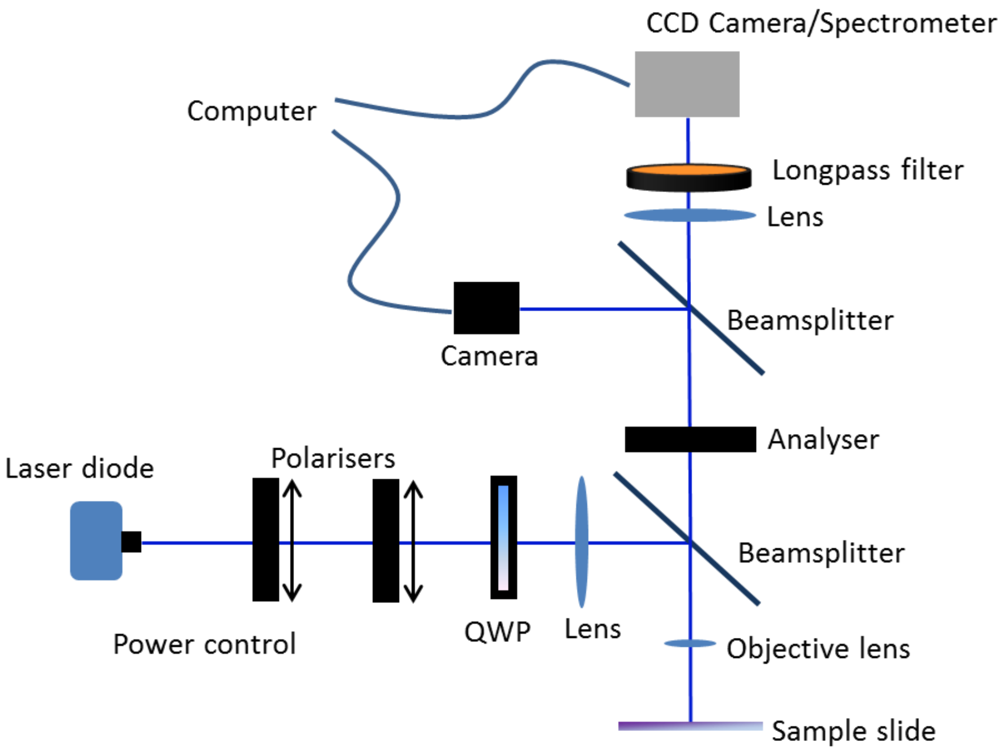

Supplementary Figure 8. Diagram of the experimental setup for luminescence measurements. The quarter-wave plate (QWP) is used to generate circularly polarised light. The two (linear) polarisers at the input after the laser diode were used to modulate the input power the first being rotated whilst the second was fixed to define the input polarisation. An optical camera with a direct view of the sample slide (designated 'Camera' in the diagram) was used to ensure that the laser was aligned to the required point on the samples. A CCD camera was used to collect luminescence signals.

Supplementary Table 1. Optical chirality density calculated from electromagnetic simulations Values shown are both for unstructured surface and meta-films, and are normalized against the values for incident CP polarization, and therefore are positive. The level of torque and hence symmetry reducing perturbation is dependent on the magnitude and not the sign of the *C* transferred.

Film type and Polarisation	*C* Incidence	*C* Transmission	*C* (Exchanged + Dissipated)
Surface LCP	0.7726	0.6912	0.0814
Surface RCP	0.7726	0.6912	0.0814
200x500 LCP	0.8235	0.7018	0.1217
200x500 RCP	0.8235	0.7062	0.1173
400x300 LCP	0.8267	0.7353	0.0914
400x300 RCP	0.8296	0.7296	0.1000
500x500 LCP	0.8245	0.726	0.0984
500x500 RCP	0.8315	0.7552	0.0763

Supplementary Table 2. Asymmetry parameters and optical chirality exchange for meta-films. An asymmetry parameter $g = \frac{2(I^{RH}-I^{LH})}{(I^{RH}+I^{LH})}$, where I^{LH} and I^{RH} are the intensities $^7F_2$ emission band and ΔIE_{RCP} - ΔIE_{LCP} is the amount of optical spin transferred to the film. The case where there is the largest transfer of optical chirality has the largest *g* factor.

Film type	*g*	ΔIE_{RCP} - ΔIE_{LCP}
200x500 LH	0.023±0.003	-0.005
200x500 RH	-0.015±0.003	0.005
400x300 LH	-0.019±0.003	0.009
400x300 RH	0.018±0.003	-0.009
500x500 LH	0.028±0.003	-0.022
500x500 RH	-0. 032±0.003	0.022
